# Oxidant–Antioxidant Status of Commercial Matcha Beverages According to Flavor Category, Preparation Medium, and Preparation Temperature: A Comparative Evaluation

**DOI:** 10.3390/foods15193410

**Published:** 2026-09-24

**Authors:** Çiler Özenir, Mihrican Çubuk, Canan Altınsoy, Duygu Ağagündüz

**Affiliations:** 1 Department of Nutrition and Dietetics, Kırıkkale University, Kırıkkale 71100, Türkiye; cileraslanalp@kku.edu.tr; 2 Department of Nutrition and Dietetics, Erzincan Binali Yıldırım University, Erzincan 24100, Türkiye; mihrican.kacar@erzincan.edu.tr; 3 Department of Nutrition and Dietetics, Recep Tayyip Erdoğan University, Rize 53100, Türkiye; 4 Department of Nutrition and Dietetics, Gazi University, Ankara 06490, Türkiye; duyguturkozu@gazi.edu.tr

**Keywords:** matcha, total antioxidant status, total oxidant status, oxidative stress index, plant-based beverages, flavored matcha, cow’s milk

## Abstract

Background: Matcha is increasingly consumed in flavored and milky forms, but the effect of preparation conditions on its antioxidant–oxidant profile remains unclear. Methods: Twenty-six commercial matcha products (unflavored and various flavors) were each evaluated under eight preparation conditions comprising four preparation media (water, cow’s milk, almond milk, and oat milk) and two temperatures (25 °C and 80 °C), yielding 208 condition-specific preparations. Thus, the eight preparations derived from each commercial product represented repeated evaluations of that product. Total antioxidant status (TAS) and total oxidant status (TOS) were measured, and the oxidative stress index (OSI) was calculated from their ratio. Temperature-related differences were evaluated using the Mann–Whitney U test, whereas differences across flavor categories, preparation media, and combined grouping variables were evaluated using the Kruskal–Wallis test; corresponding effect sizes were reported. Results: Only TAS and TOS differed significantly by flavor type (*p* < 0.001). Commercial products categorized as beetroot-flavored showed the lowest TAS and TOS values; these findings reflect differences among the commercial formulations evaluated and should not be interpreted as an independent effect of beetroot flavoring. TAS, TOS, and OSI differed significantly by preparation medium (*p* < 0.001), with large effect sizes (ε^2^ = 0.557, 0.637, and 0.638 for TAS, TOS, and OSI, respectively). Temperature differed significantly across all three parameters. The highest TAS was observed in samples prepared with water at 25 °C; the lowest TOS and OSI values were observed in samples prepared with cow’s milk at 25 °C. Among the combined-group comparisons, the largest effect sizes were observed for the combined temperature–preparation medium groups (ε^2^ = 0.654–0.746) and combined preparation medium–flavor category groups (ε^2^ = 0.723–0.780). Conclusions: The assay-detectable oxidant–antioxidant profile of the commercial matcha beverages varied across the preparation conditions and commercial product categories evaluated. These findings represent matrix- and assay-dependent analytical differences and should not be interpreted as evidence of physiological oxidative stress or health effects.

## 1. Introduction

Matcha is a traditional Japanese powdered green tea produced by finely grinding tencha obtained from specially cultivated leaves of *Camellia sinensis* (L.) Kuntze [1,2]. Unlike conventional green-tea infusions, in which the leaves are removed after brewing, matcha is consumed as a suspension containing the ground leaf material [2,3]. It therefore contains a complex mixture of catechins and other phenolic compounds, chlorophylls, caffeine, L-theanine, and additional leaf constituents [1,2,3]. Preharvest shading is a defining cultivation practice that modifies this phytochemical profile, particularly by increasing chlorophyll and L-theanine concentrations, and may thereby contribute to the distinctive bioactive composition and antioxidant-related properties of matcha [1,3]. Together with increasing interest in plant-derived and functional beverages, these characteristics have contributed to the expanding use of matcha in beverages and other food formulations [3,4].

The abundance of catechins, particularly epigallocatechin gallate, and other redox-active compounds has prompted considerable interest in the in vitro antioxidant characteristics of matcha-based beverages [1,2]. However, characterization based exclusively on a single antioxidant-capacity assay provides only a partial description of the chemical response of a complex beverage. Total antioxidant status (TAS) represents the cumulative antioxidant response detectable under the specified assay conditions, whereas total oxidant status (TOS) reflects the collective oxidizing capacity detected by the corresponding colorimetric reaction. The oxidative stress index (OSI), calculated from the ratio of TOS to TAS after unit adjustment, provides a ratio-based summary of these two measurements but is not an independent analytical endpoint [5,6]. Previous studies applying TAS, TOS, and OSI to tea and herbal-tea infusions have reported variation according to tea type, product formulation, brand, and preparation protocol [7,8]. A joint evaluation of these parameters may, therefore, provide complementary information about the in vitro oxidant–antioxidant profiles of matcha formulations.

Preparation with milk or milk-like beverages may further modify the measured chemical characteristics of matcha through food-matrix interactions [2,4]. Cow’s milk contains caseins and whey proteins that can associate with catechins and other tea polyphenols through hydrogen bonding, hydrophobic interactions, and other noncovalent forces [9,10]. In a heated green-tea–milk model, increasing milk-protein concentration enhanced phenol–protein interactions and reduced the ultrafilterable unbound polyphenol fraction, which was accompanied by a decrease in antioxidant activity [11]. However, the analytical consequences of these interactions are method-dependent. Bourassa et al. (2013), for example, reported that the influence of α-casein on the antioxidant activity of tea polyphenols varied according to the polyphenol structure and the analytical method used [12]. Milk–polyphenol interactions may therefore alter assay-detectable antioxidant responses without necessarily demonstrating polyphenol degradation or reduced physiological availability. In addition to cow’s milk, plant-based milk alternatives are increasingly used in matcha-based beverages, particularly among consumers with lactose intolerance or milk allergy, those following plant-based diets, and those with environmental or ethical concerns. These products are not compositionally equivalent to cow’s milk or to one another, because their protein, lipid, carbohydrate, fiber, mineral, and added-ingredient profiles depend on both the source material and commercial formulation [13,14,15]. Plant-based milk alternatives differ substantially in their nutritional and phytochemical composition. Soy beverages generally resemble cow’s milk more closely in protein content, whereas almond, oat, rice, and coconut beverages tend to contain less protein [13,16]. Their phenolic profiles also vary according to botanical source, with characteristic isoflavones occurring in soy beverages and hydroxycinnamic acids and avenanthramides in oat beverages [15,17]. Accordingly, plant-based beverages should be regarded as compositionally distinct food matrices that may influence the assay-detectable oxidant–antioxidant profile of matcha.

Commercial flavored matcha formulations introduce an additional level of compositional heterogeneity. Depending on the product, flavoring preparations, fruit-derived ingredients, sweeteners, pigments, and other formulation components may alter pH, color, phenolic composition, and in vitro antioxidant measurements [4,18]. Recent studies comparing pure matcha with strawberry- or mixed-berry-containing formulations have indicated that fruit incorporation may modify physicochemical composition, including pH and phenolic, flavonoid, anthocyanin, and vitamin C levels, together with color, sensory characteristics, and antioxidant activity [19,20]. Studies of tea-based beverages have likewise indicated that the effects of milk, sugar, honey, and other added ingredients on measured antioxidant activity vary according to ingredient type and concentration, tea type, and analytical method [21,22]. Flavor type should therefore be considered not only a sensory characteristic but also a marker of potentially different commercial formulations.

Preparation temperature is another important determinant of the measured composition of matcha beverages [2]. Increasing temperature can enhance mass transfer and the release of catechins and other soluble constituents, although the response depends on the individual compound and exposure duration [23]. Studies directly examining matcha at different temperatures have also reported temperature-dependent changes in total phenolic, flavonoid, and vitamin C contents, as well as antioxidant activity, although the direction and magnitude of these responses differed according to the product, preparation procedure, and analytical approach [24,25]. Conversely, sufficiently intense or prolonged thermal exposure may promote catechin epimerization, oxidation, or degradation, with catechin stability additionally influenced by pH, oxygen availability, concentration, and matrix composition [26].

Previous studies of matcha and matcha-based formulations have evaluated selected bioactive components and antioxidant properties using assays reflecting different reaction mechanisms, including radical-scavenging and reducing activities [24,25]. More recent studies have further demonstrated that fruit incorporation and extraction temperature can modify the measured phenolic composition and antioxidant characteristics of matcha-based formulations [19,20,27]. However, limited information is available on the combined influence of flavor category, preparation medium, and temperature on the overall antioxidant–oxidant balance of commercially available matcha beverages. Although TAS, TOS, and OSI have been applied in studies of tea infusions, their use in matcha beverages remains limited [7,8]. Importantly, TAS and TOS provide complementary information on assay-detectable antioxidant and oxidant capacities, respectively, whereas OSI is a derived index that reflects the relative relationship between these two measurements. This distinction is relevant because previous studies of tea and other complex beverage matrices have shown that antioxidant and oxidant capacities may change concurrently and do not necessarily produce a corresponding change in OSI [7,8,28,29,30]. To the best of our knowledge, no previous study has simultaneously evaluated unflavored and flavored commercial matcha products prepared in water, cow’s milk, almond milk, and oat milk at different temperatures using TAS, TOS, and OSI. Therefore, the present study aimed to characterize and compare the assay-detectable oxidant–antioxidant profiles of 26 commercial matcha products according to commercial flavor category, preparation medium, and temperature. The products were prepared with water, whole cow’s milk, almond milk, or oat milk at 25 °C and 80 °C. It was hypothesized that commercial product formulation and preparation conditions would be associated with distinct TAS, TOS, and OSI profiles.

## 2. Materials and Methods

A total of 26 commercial matcha products were included in the study. These comprised one unflavored and eight flavored categories from six brands. The unflavored products contained matcha as the primary ingredient without added flavoring ingredients, whereas the flavored products contained matcha together with additional flavoring and/or other ingredients as specified in their product formulations. Detailed information on the product categories and their ingredient compositions is provided in Table 1. The products were purchased from online marketplaces in Türkiye between May and June 2026. Flavor categories were selected according to market availability during the study period, and only flavors represented by products from at least two different commercial brands were included. To minimize the potential influence of storage duration on the analytical results, only products with a manufacturer-declared remaining shelf life of at least one year at the time of purchase were selected.

Each product was prepared at two temperatures, 25 and 80 °C, using four preparation media: water, whole cow’s milk, almond milk, and oat milk. Almond and oat milks were selected as widely accepted plant-based alternatives representing nut- and cereal-based matrices, respectively [31]. This experimental design yielded eight preparation conditions for each product and a total of 208 matcha preparations.

Temperatures of 25 °C and 80 °C were selected to represent room-temperature and hot preparation conditions, respectively. Their selection was based on the preparation instructions provided on the product labels and on temperatures used in previous analytical studies of matcha infusions [24,32]. This design enabled the evaluation of the potential effects of preparation temperature on the antioxidant and oxidative characteristics of the matcha products.

The label information provided on the packaging of the matcha products included in the study was examined in detail. Products were classified as unflavored or flavored based on the ingredient information declared by the manufacturers. Unflavored products contained 100% matcha, whereas flavored products contained matcha together with additional flavoring ingredients or flavoring components, where declared. Information on flavor variety, brand, declared percentages of matcha and flavoring components, natural flavoring declarations, additives, product quality claims, country of origin, and packaging type was recorded. Detailed label-based compositional characteristics of the 26 products are presented in Table 1.

A 2% (*w*/*v*) matcha concentration was used for all preparations, achieved by combining 1.0 g of matcha powder with sufficient preparation liquid to obtain a final volume of 50 mL. This concentration was selected with reference to the preparation instructions provided on the product labels and was broadly comparable to concentrations used in previous analytical studies of matcha dispersions and infusions [24,33]. A final volume of 50 mL was selected as a practical laboratory-scale volume that allowed accurate and reproducible weighing of 1.0-g matcha portions while maintaining the predetermined concentration.

For water-based preparations, 1.0 g of matcha powder was combined with 50 mL of water. For milk-based, latte-style preparations, 1.0 g of matcha powder was first combined with 12 mL of water. Direct addition of matcha powder to the milk matrices did not provide adequate dispersion. Subsequently, 38 mL of whole cow’s milk, almond milk, or oat milk was added. For preparations assigned to 80 °C, the water- or milk-based preparation medium was first heated to 80 °C using a temperature-controlled heating device, and matcha was then added. The temperature did not exceed 80 °C in any preparation. The samples were subsequently shaken for 10 min, during which they were allowed to cool naturally; thus, 80 °C represents the initial preparation temperature rather than a temperature maintained throughout the shaking period. Preparations assigned to 25 °C were prepared using media adjusted to 25 °C. The water-to-milk ratio was established according to the preparation instructions on the product labels and applied consistently across all milk matrices. All shaking was performed on a laboratory shaker (DLAB Orbital Shaker, SK-O180-Pro; DLAB Scientific Co., Ltd., Beijing, China) for 10 min at 180 rpm. Before the TAS and TOS analyses, plant particles were separated from all samples through filtration. Sterile 0.45 µm PTFE syringe filters (25 mm diameter; ISOLAB, Eschau, Germany, Cat. No. 094.05.002) were used. The 0.45-µm filtration step was selected as a practical compromise to obtain particle-reduced samples compatible with automated analysis while preserving the soluble beverage fraction as much as practicable. Nevertheless, filtration may have removed suspended particulate material and some matrix-associated constituents or complexes. Therefore, the subsequent TAS and TOS measurements represent the filtered fraction of the prepared beverages rather than the complete matcha suspension as consumed.

The preparation media consisted of a commercially available full-fat UHT cow’s milk, a barista-style almond beverage, and a barista-style oat beverage. The almond and oat beverages were obtained from the same manufacturer, whereas the cow’s milk was obtained from a different manufacturer. According to the product labels, the cow’s milk contained 3.5 g fat, 4.5 g carbohydrate (4.5 g sugars), and 3.0 g protein per 100 mL. The almond beverage contained 1.2 g fat, 2.5 g carbohydrate (2.5 g sugars), and 0.5 g protein per 100 mL, whereas the oat beverage contained 3.1 g fat, 6.7 g carbohydrate (3.3 g sugars), and 0.8 g protein per 100 mL. The major added ingredients of the plant-based beverages included sugars, vegetable oil, fiber, pea protein, mineral salts, stabilizers, and vitamins, as applicable according to the product labels. The same product lot/batch of each preparation medium was used throughout the study. Medium-only controls (water, whole cow’s milk, almond milk, and oat milk) were not analyzed for TAS and TOS; therefore, the measured values represent the overall assay responses of the complete matcha beverage formulations, including potential contributions from the preparation media themselves.

Immediately after preparation, each beverage sample was analyzed in duplicate for TAS and TOS using two aliquots from the same preparation (technical analytical duplicates; *n* = 2). Each beverage condition was prepared once. Thus, the 208 beverage preparations were analyzed in duplicate for each assay, yielding 416 replicate measurements per assay. The mean of the two duplicate measurements for each preparation was calculated and used as the single TAS and TOS value for that beverage condition in the statistical analyses. The products’ descriptive characteristics and the ingredients declared on their labels were recorded, while TAS, TOS, and OSI were determined by laboratory analyses.

### 2.1. Total Antioxidant Status (TAS) (mmol/L)

Total antioxidant status (TAS) was measured using a commercially available assay kit (Rel Assay Diagnostics, Gaziantep, Türkiye) on a Mindray BS-400 automated chemistry analyzer (Mindray Bio-Medical Electronics Co., Shenzhen, China) according to the manufacturer’s instructions. Following analyzer calibration, two-level quality-control materials were analyzed before sample measurements. This method is based on the bleaching of the characteristic color of a more stable ABTS [2,2′-azino-bis (3-ethylbenzothiazoline-6-sulfonic acid)] radical cation by antioxidants [6].

The change in absorbance at 660 nm is proportional to the total antioxidant capacity of the sample. Calibration was performed using Trolox, a water-soluble vitamin E analogue. The results were expressed as mmol Trolox equivalents/L. For each analysis, 300 µL of Reagent 1 and 18 µL of sample were mixed automatically, and the initial absorbance was measured at 660 nm after 30 s. Subsequently, 45 µL of Reagent 2 was added, followed by incubation for 5 min; the final absorbance was measured at 660 nm. No additional sample-specific blanking, dilution-linearity testing, recovery experiments, or matrix-specific validation procedures were performed for the beverage matrices evaluated in the present study.

### 2.2. Total Oxidant Status (TOS) (µmol/L)

Total oxidant status (TOS) was measured using a commercially available assay kit (Rel Assay Diagnostics, Gaziantep, Türkiye) on a Mindray BS-400 automated chemistry analyzer (Mindray Bio-Medical Electronics Co., Shenzhen, China) according to the manufacturer’s instructions. Following analyzer calibration, two-level quality-control materials were analyzed before sample measurements. According to the manufacturer’s analytical procedure, bichromatic measurement using a secondary wavelength was applied to reduce interference from intrinsic sample color and background absorbance. The assay is based on the oxidation of the ferrous ion–o-dianisidine complex to ferric ions by oxidants present in the sample. The oxidation reaction is enhanced by amplifying molecules present in the reaction medium. Ferric ions subsequently form a colored complex with a chromogen in an acidic medium, and the color intensity, measured spectrophotometrically at 530 nm, is proportional to the total oxidant status of the sample. The assay was calibrated using hydrogen peroxide, and the results were expressed as micromoles of hydrogen peroxide equivalent per liter (μmol H_2_O_2_ equiv./L) [5].

For each analysis, 300 µL of Reagent 1 and 45 µL of sample were mixed automatically, and the initial absorbance was measured at 530 nm after 30 s. The use of a pre-reaction absorbance reading for sample-background correction and bichromatic measurement with a secondary wavelength is consistent with the analytical principle of the automated TOS method described by Erel [5]. Subsequently, 15 µL of Reagent 2 was added, followed by incubation for 5 min, and the final absorbance was measured at 530 nm [5]. No additional sample-specific blanking, dilution-linearity testing, recovery experiments, or matrix-specific validation procedures were performed for the beverage matrices evaluated in the present study.

### 2.3. Oxıdative Stress Index (OSI)

The oxidative stress index (OSI) was calculated as the ratio of TOS to TAS. For this calculation, TAS values were converted to μmol/L, and OSI was calculated using the following formula: OSI (arbitrary unit) = TOS (μmol H_2_O_2_ equivalent/L)/TAS (μmol Trolox equivalent/L) [34,35,36].

### 2.4. Statistical Analysis

Data from the study were analyzed using SPSS version 22.0. The normality of the variables was assessed using both visual methods (histograms and probability plots) and analytical methods (Kolmogorov–Smirnov and Shapiro–Wilk tests). Categorical variables are presented as frequency (*n*) and percentage (%), while quantitative variables are presented as range, mean, and standard deviation ($\bar{X}$ ± SD). The Pearson chi-square test was used to compare categorical variables. Since the continuous variables did not follow a normal distribution, the Kruskal–Wallis test was used to assess differences between groups, followed by pairwise comparisons using the Mann–Whitney U test. The Bonferroni correction was applied to control the risk of Type I error arising from multiple pairwise comparisons. In all statistical tests, a 95.0% confidence interval was used, and statistical significance was set at *p* < 0.05. Jamovi version 2.7.4 was used to calculate the effect sizes. For effect size estimation, rank-biserial correlation was reported for the Mann–Whitney U test, whereas epsilon-squared (ε^2^) values were reported for the Kruskal–Wallis H test. Rank-biserial correlation values of 0.10–0.29 were interpreted as indicating a small/weak effect, values of 0.30–0.49 as indicating a moderate effect, and values > 0.50 as indicating a large/strong effect. For epsilon-squared, values of 0.01–0.05 were interpreted as indicating a small/weak effect, values of 0.06–0.14 as a moderate effect, and values > 0.14 as a large/strong effect [37,38].

## 3. Results

### 3.1. TAS, TOS, and OSI Values of Matcha Teas According to Flavor Type

The total antioxidant status (TAS), total oxidant status (TOS), and oxidative stress index (OSI) values for matcha teas categorised by flavor are presented in Table 2. Statistically significant differences in TAS and TOS values were found between the flavor varieties (*p* < 0.001 and *p* = 0.001, respectively). The highest mean TAS value was found in lemon-flavored matcha teas (4.51 ± 0.08 mmol/L), whilst the lowest was observed in beetroot-flavored matcha teas (2.73 ± 1.21 mmol/L). Similarly, the highest mean TOS value was found in lemon-flavored matcha teas (103.08 ± 68.43 µmol/L), whilst the lowest TOS value was observed in beetroot-flavored matcha teas (25.17 ± 25.37 µmol/L). No statistically significant differences in OSI values were found among the flavor varieties (*p* = 0.263). OSI averages ranged from 1.16 ± 1.05 to 2.29 ± 1.52, with the lowest value observed in beetroot-flavored matcha teas and the highest in lemon-flavored matcha teas.

### 3.2. TAS, TOS, and OSI Values of Matcha Teas According to Preparation Medium and Temperature

Statistically significant differences were observed among the eight preparation conditions in terms of TAS, TOS, and OSI values (*p* < 0.001 for all; Table 3). Descriptively, the highest mean TAS value was observed in the samples prepared with water at 25 °C (4.55 ± 0.06 mmol/L), whereas the lowest mean value was observed in those prepared with cow’s milk at 80 °C (3.79 ± 0.89 mmol/L). The highest mean TOS and OSI values were recorded in the samples prepared with oat milk at 80 °C (205.81 ± 41.49 µmol/L and 4.64 ± 0.77, respectively). The lowest mean TOS and OSI values were observed in the samples prepared with cow’s milk at 25 °C (30.44 ± 7.26 µmol/L and 0.70 ± 0.15, respectively) (Table 3).

Table 4 presents the TAS, TOS, and OSI values of matcha teas prepared across the evaluated flavor categories, preparation media, and temperatures. Across most non-beetroot flavor categories, preparations with cow’s milk generally showed lower TOS and OSI values, whereas oat-beverage preparations showed the highest values. Almond-beverage preparations generally showed intermediate-to-high TOS and OSI values, particularly at 80 °C.

Differences across temperatures were more pronounced for TOS and OSI than for TAS. In particular, TOS and OSI values in almond- and oat-beverage preparations were generally higher at 80 °C than at 25 °C, whereas differences in water- and cow’s-milk preparations were less consistent. The most pronounced increases were observed in several almond-beverage preparations, while oat-beverage preparations generally maintained the highest absolute TOS and OSI values at 80 °C. TAS varied comparatively less across temperatures.

The statistical comparisons among preparation media further supported these descriptive patterns. At both 25 °C and 80 °C, significant differences among preparation media were observed for TOS and OSI in most non-beetroot flavor groups, with oat milk generally showing the highest and cow’s milk the lowest values.

The beetroot-flavored samples exhibited a different profile from the other flavor groups; their TAS, TOS, and OSI values were markedly lower overall, particularly in water-based preparations, and the differences among preparation media were smaller and generally not statistically significant. The preparation temperature did not result in a statistically significant difference in the beetroot-flavored samples.

### 3.3. Differences in TAS, TOS, and OSI According to Temperature, Flavor Type, Preparation Medium, and Combined Groups

Differences in TAS, TOS, and OSI according to temperature, flavor type, preparation medium, and combined grouping variables are presented in Table 5. Significant differences across temperatures were observed in TAS, TOS, and OSI; effect sizes were small for TAS and TOS and moderate for OSI. Flavor type was associated with significant differences in TAS and TOS, with large and moderate effect sizes, respectively, whereas OSI did not differ significantly across flavor categories. Significant differences were also observed across preparation media for all three parameters, with large effect sizes.

When temperature and flavor type, temperature and preparation medium, and preparation medium and flavor type were combined to define multi-level grouping variables, significant differences were observed among the resulting groups for TAS, TOS, and OSI. These analyses represent comparisons among combined groups and should not be interpreted as tests of statistical interaction between the corresponding factors. The largest effect sizes among these combined-group comparisons were observed for the temperature–preparation medium groups (ε^2^ = 0.654–0.746) and preparation medium–flavor type groups (ε^2^ = 0.723–0.780).

## 4. Discussion

In recent years, matcha has gained increasing popularity as a beverage consumed in a wide variety of flavored formulations and prepared with water or different milk-based media [4,18,27,39]. Despite this growing diversity, the extent to which product formulation and preparation conditions influence its measured oxidant–antioxidant characteristics remains unclear. In the present study, TAS and TOS differed significantly among flavor groups, whereas OSI did not. Significant differences in all three parameters were observed with respect to preparation medium and temperature. Differences among preparation medium–temperature combinations were also pronounced, with particularly high TOS and OSI values in almond- and oat-beverage samples prepared at 80 °C. Overall, these findings indicate that the measured oxidant–antioxidant profiles of commercial matcha beverages varied according to product formulations and preparation conditions.

The flavor-related differences were largely attributable to the commercial products categorized as beetroot-flavored, which exhibited lower TAS and TOS values than the other flavor categories. No significant differences were observed among the remaining flavor categories, and OSI likewise did not differ significantly by flavor. Because both TAS and TOS were lower in the commercial products categorized as beetroot-flavored, the lower TOS value should not be interpreted independently as evidence of a more favorable oxidant–antioxidant profile. Although beetroot contains betalains and phenolic compounds with recognized antioxidant properties, the present findings cannot be attributed specifically to beetroot or generalized to the inherent antioxidant potential of beetroot-derived compounds [40,41]. Importantly, the study compared heterogeneous commercial formulations rather than standardized matcha–beetroot mixtures. Matcha content was not declared for eight flavored products; among products with quantitative labeling, matcha content ranged from 80.0% to 99.5% and flavoring content from 0.5% to 20.0% (Table 1). Therefore, the observed differences among flavor categories cannot be attributed specifically to the flavoring ingredients. Consequently, preparations made using the same nominal amount of commercial product did not necessarily contain the same amount of matcha, introducing an additional source of confounding in flavor-category comparisons. Accordingly, the flavor-related findings should be interpreted as differences among the commercial formulations evaluated rather than as flavor-specific effects. Rather, they should be interpreted as differences among the commercial formulations evaluated, which varied simultaneously in matcha proportion, flavoring concentration, additional ingredients, brand-specific formulation, processing, storage history, and other product-level characteristics. This interpretation is consistent with previous evidence showing substantial variation in catechin composition, phenolic content, and measured antioxidant activity among commercially available green tea and matcha products [42,43]. Although betalain stability is known to be affected by temperature, oxygen, light, and pH [41,44,45,46], betalain concentrations and degradation products were not measured in the present study; therefore, degradation of beetroot-derived pigments cannot be established as an explanation for the observed findings. Label information further demonstrated the heterogeneity of the commercial products. Approximately 62% of flavored products declared natural flavorings, although their composition and concentration were not specified. Additives were declared in only three products and consisted of tricalcium phosphate (Table 1). Manufacturer-declared quality claims and packaging characteristics were recorded descriptively but were not evaluated as independent analytical factors. Consequently, their contribution to the measured TAS, TOS, and OSI values could not be determined.

The analysis of the eight preparation conditions, comprising four preparation media (water, cow’s milk, oat milk, and almond milk), each evaluated at two temperatures (25 °C and 80 °C), revealed significant differences in TAS, TOS, and OSI. Samples prepared with water showed the highest TAS values at both temperatures, whereas those prepared with cow’s milk exhibited the lowest TOS values. Although cow’s-milk preparations also showed the lowest OSI values, their post hoc groupings overlapped with those of some water and almond-milk conditions. In contrast, the highest TOS and OSI values were observed in oat milk at 80 °C. In almond milk, both parameters were also significantly higher at 80 °C than at 25 °C, while TAS changed only slightly. A similar temperature-associated increase in TOS and OSI was observed in oat milk. These findings indicate that the measured oxidant–antioxidant profile varied substantially across preparation conditions. These findings indicate that the overall oxidant–antioxidant profile differed substantially among the complete beverage formulations. However, medium-only controls were not analyzed in the present study. Therefore, the observed differences in TAS, TOS, and OSI cannot be attributed specifically to interactions between matcha constituents and the respective preparation media, as the intrinsic antioxidant and oxidant characteristics of the media themselves may also have contributed to the measured responses. Accordingly, the results should be interpreted as differences in the overall assay responses of the complete matcha beverage formulations. Consistent with these condition-specific observations, significant differences were detected among combinations of temperature and preparation medium for TAS, TOS, and OSI, with large effect sizes for all three parameters. However, because these comparisons evaluated the eight combined preparation groups rather than a formal factorial interaction term, the differing temperature-associated patterns across beverage matrices should not be interpreted as evidence of a statistically confirmed temperature × preparation medium interaction. The lower TAS observed in cow’s-milk preparations, particularly at 80 °C, may partly reflect the intrinsic characteristics of the milk matrix and/or interactions between matcha-derived polyphenols and dairy proteins. However, this interpretation remains hypothetical because protein binding, free catechin concentrations, and individual catechins were not directly measured in the present study. Green-tea catechins can associate with milk proteins, including caseins, through hydrophobic and other noncovalent interactions [47]. Because the Erel TAS assay is based on the decolorization of the ABTS radical cation by antioxidant constituents, protein–polyphenol interactions and other matrix effects could influence the measured TAS response [6,12,48]. However, protein binding, free catechin concentrations, and individual catechins were not measured in the present study; therefore, this mechanism remains hypothetical, and the lower TAS cannot be interpreted as evidence of a reduction in total catechin content. Additional evidence indicates that antioxidant responses in dairy matrices may be non-additive. Demirci Çekiç et al. (2015) [48] reported deviations from additivity in ABTS-based measurements when milk protein fractions were incorporated into a milk matrix, whereas the corresponding CUPRAC measurements were less affected. Although these assays are not identical to the Erel TAS method, the findings illustrate that protein-rich dairy matrices can influence assay-dependent antioxidant responses [48]. Accordingly, the TAS measured in the complete cow’s-milk–matcha preparation cannot be attributed solely to freely available matcha-derived antioxidants. Supporting the plausibility of an assay-dependent protein effect, α-casein has been reported to reduce the ABTS-detectable antioxidant activity of individual tea polyphenols, although the magnitude of this effect differed among compounds and analytical methods [12]. More directly, Alhazmy et al. (2025) [27] compared matcha prepared with cow’s milk and several plant-based beverages and reported lower total phenolic and flavonoid contents, radical-scavenging activity, metal-chelating activity, and ferric-reducing power after milk addition. Cow’s milk produced the largest, or among the largest, reductions across several analytical endpoints, whereas the effects of almond- and oat-based beverages were generally less pronounced. These findings are consistent with the lower assay-detectable TAS observed in the cow’s-milk preparations in the present study. However, Alhazmy et al. evaluated different antioxidant-related assays under a single preparation-temperature condition and did not measure TAS, TOS, OSI, or protein–polyphenol binding directly; therefore, their findings provide comparative support rather than a direct mechanistic explanation for the present results [27].

Jurupan and Bumrungpert (2026) [39] reported the highest oxygen radical absorbance capacity in cow’s-milk matcha latte and lower values in soy-, oat-, almond-, and water-based preparations. However, the latter study used ORAC as the sole antioxidant-capacity assay, included only one water-based formulation, and did not analyze milk-only controls. Differences in preparation protocols and in the intrinsic antioxidant contribution of the preparation media may also have affected the measured responses. These divergent findings indicate that the apparent effect of milk addition cannot be generalized across analytical methods and that the lower TAS observed in the cow’s-milk preparations in the present study should be interpreted as an assay- and matrix-specific response rather than as evidence of a universal reduction in antioxidant potential [39]. Chen et al. (2024) [11] reported that increasing milk-protein concentration in a heated green-tea model increased phenol–protein binding and decreased the free-polyphenol fraction and DPPH and FRAP responses. However, all milk–tea treatments in that study were heated at 100 °C for 15 min; thus, the study demonstrates a protein-concentration effect under a common heating condition but does not independently establish that heating itself increased protein–polyphenol binding. More direct temperature-dependent evidence has been obtained in a β-lactoglobulin–EGCG model, in which heating increased binding affinity and treatments above 85 °C reduced ABTS and FRAP responses [49]. Nevertheless, this simplified model is not directly equivalent to a whole-milk–matcha preparation at 80 °C. Previous studies indicate that milk and plant-based beverages may themselves contribute to the measured oxidant–antioxidant profile. Measurable TAS, TOS, and OSI values have been reported for cow’s milk, and differences in these parameters have also been demonstrated among cow’s milk and plant-based beverages, including almond- and oat-based products (Ağagündüz, 2021; Kala, 2024; Özenir et al., 2025) [28,50,51]. These findings indicate that the preparation media cannot be considered analytically inert matrices. Therefore, because medium-only controls were not included in the present study, the intrinsic contribution of each preparation medium could not be separated from the TAS, TOS, and OSI responses measured in the complete matcha beverages. This limitation is further compounded by the fact that each preparation-medium category was represented by a single commercial product and that the plant-based beverages contained several additional formulation components. Accordingly, the observed differences should be interpreted as specific to the evaluated commercial formulations and should not be generalized to cow’s milk, almond beverages, or oat beverages as entire food categories.

Heat-related changes in tea catechins, including epimerization, degradation, hydrolysis, and oxidation or condensation reactions, may have contributed to the temperature-dependent TAS differences observed in the present study [52,53,54]. However, these mechanisms have largely been demonstrated in aqueous catechin systems or tea infusions exposed to controlled and, in some cases, relatively prolonged thermal treatments. Their direct applicability to the present study is therefore limited because matcha was prepared by briefly mixing the powder with water- or milk-based media rather than by conventional infusion, and the resulting preparations were filtered before biochemical analysis. Moreover, changes in catechin composition do not necessarily result in proportional changes in assay-measured antioxidant capacity. In a simplified aqueous EGCG system, temperature- and pH-dependent degradation and epimerization occurred without significant corresponding changes in total antioxidant capacity measured using radical-based assays [55]. In the present study, the TAS response may also have been influenced by the surrounding matrix. Interactions between tea polyphenols and milk proteins can reduce the freely detectable polyphenol fraction and modify the measured antioxidant response [6,12,49]. Such effects may be particularly relevant to matcha because the powdered leaves were dispersed directly into the milk matrix before filtration, allowing matrix-dependent interactions during sample preparation. Accordingly, the observed TAS differences cannot be attributed solely to changes in catechin concentration or thermal transformation. Protein–polyphenol interactions, differences in individual catechin composition, the presence of suspended matcha particles, and matrix-related effects on the ABTS-based TAS assay may have contributed. Because these factors were not measured directly, they should be regarded as plausible mechanisms rather than established causes.

The temperature-associated TAS pattern may reflect competing effects of heating on the dispersion, solubilization, and assay accessibility of matcha-derived antioxidant constituents, and their concurrent chemical transformation. TAS changed only modestly between 25 and 80 °C in the water-, almond-milk-, and oat-milk-based preparations, whereas a more pronounced decline was observed in the cow’s-milk-based preparations. By contrast, TOS and the derived OSI values were higher at 80 °C in almond- and oat-milk-based preparations, while the corresponding changes in water were relatively limited (Table 4). Consistent with these patterns, the overall temperature-related difference was smaller for TAS than for TOS and OSI, whereas differences among the combined temperature–preparation medium groups showed large effect sizes for all three parameters, particularly for TOS and OSI (Table 5). These findings indicate substantial differences among the combined preparation groups; however, the analysis did not formally test a temperature × preparation medium interaction. The available literature on matcha preparation and extraction primarily addresses phenolic recovery, catechin composition, and antioxidant capacity measurements rather than TOS, OSI, or defined oxidation products. Therefore, it provides a more direct basis for interpreting the TAS pattern than for explaining the higher TOS and OSI values.

In a response-surface study conducted directly with matcha, extraction temperature, duration, and liquid-to-solid ratio jointly influenced phenolic recovery and antioxidant-assay responses, with a combined optimum of 80 °C for 20 min under the heat-assisted extraction conditions examined [25]. In beverage-like matcha preparations, Jakubczyk et al. (2020) reported generally higher DPPH and FRAP responses at elevated preparation temperatures, although the temperature associated with the highest total polyphenol content differed between the two commercial matcha products examined [24]. More recent studies of pure and fruit-containing matcha formulations have likewise reported compound- and assay-dependent responses across extraction temperatures [19,20]. However, because the cold extractions in these studies lasted 12 h whereas the heated extractions lasted 5 min, the independent effect of temperature could not be distinguished from the effect of extraction duration. Direct evidence also indicates that the liquid matrix can modify the measured antioxidant capacity of matcha beverages. Jurupan and Bumrungpert (2026) reported significant differences in ORAC values among matcha lattes prepared with cow’s milk, soy milk, oat milk, and almond milk and a water-based matcha control [39]. Although these results cannot be directly compared with the present TAS values because of differences in products, preparation conditions, and analytical methods, they support the interpretation that measured antioxidant responses may differ across preparation matrices. These findings indicate that temperature-associated changes in total antioxidant measurements cannot be assumed to correspond uniformly to changes in total phenolic content or individual catechin concentrations. They also support interpreting the present TAS results as matrix- and method-dependent responses of whole matcha preparations, rather than as direct measures of catechin concentration. Consistent with this interpretation, the magnitude of the overall temperature-related difference was smaller for TAS than for either TOS or OSI; differences among the combined temperature–preparation medium groups showed large effect sizes for all three parameters, with the largest effect size observed for OSI (Table 5). In contrast, the higher TOS and corresponding OSI values observed at 80 °C in the almond- and oat-beverage preparations are not readily explained by the available matcha literature and therefore warrant cautious interpretation. The Erel TOS assay reflects the collective oxidizing capacity detected in the sample rather than any specific oxidation product; consequently, the present findings cannot be attributed specifically to catechin oxidation, hydrogen peroxide formation, lipid oxidation, or another defined oxidative pathway [5]. Moreover, medium-only controls were not included, preventing separation of the intrinsic TOS contribution of the commercial almond and oat milk from the response measured in the complete matcha preparations. Future studies should therefore incorporate medium-only controls and targeted measurements of H_2_O_2_, lipid oxidation products, individual catechins, and pH to clarify the origin of the elevated TOS response.

The elevated TOS and corresponding OSI values observed in the almond- and oat-milk-based preparations should be interpreted cautiously, because these commercial plant-based milks are compositionally complex colloidal matrices rather than inert diluents. In green-tea-extract–oat-milk model systems, oat-milk fat content influenced polyphenol binding, physical stability, and gastrointestinal polyphenol bioaccessibility [56]. A subsequent in vitro digestion study similarly showed that the oat-milk matrix and its fat content affected macronutrient digestion and the bioaccessibility of tea polyphenols [57]. These findings demonstrate matrix-dependent behavior in oat-based tea systems, but cannot be extrapolated directly to almond milk or the present matcha preparations. Moreover, neither study evaluated preparation temperature, TOS, OSI, or specific oxidation products and therefore cannot directly explain the higher values observed at 80 °C. In the present study, differences in TOS and OSI across preparation media showed large effect sizes, while differences among the combined temperature–preparation medium groups showed even larger effect sizes, particularly for OSI (Table 5). These findings indicate that oxidant-related assay responses differed substantially across the evaluated preparation conditions, although they do not establish a formal temperature × preparation medium interaction or the specific mechanisms underlying these differences.

A temperature-related contribution from tea constituents is nevertheless chemically plausible. Tea catechins and tea extracts can undergo molecular-oxygen-dependent autoxidation, although the rate and extent of this process depend on factors such as pH, polyphenol concentrations, and oxygen availability [58]. Temperature-dependent hydrogen peroxide formation has also been demonstrated in EGCG and tea-beverage model systems [59]. However, these studies were conducted under experimental conditions that differed from the short-duration matcha preparation protocol used here, in which samples were filtered prior to biochemical analysis. Therefore, their findings provide mechanistic context rather than direct evidence for the source of the elevated TOS values. The Erel TOS assay measures the collective capacity of oxidizing species in a sample to oxidize ferrous ions to ferric ions under acidic conditions, with the results expressed as H_2_O_2_ equivalents [5]. The comparatively larger differences in TOS and OSI across temperatures, together with the large effect sizes observed among the combined temperature–preparation medium groups (Table 5), indicate substantial variation in oxidant-related assay responses across the evaluated conditions. Because the TOS assay reflects overall oxidizing capacity rather than a specific oxidant compound or lipid peroxidation product, elevated TOS values cannot be attributed to a particular oxidation mechanism.

Because OSI was calculated as the ratio of the measured TOS to TAS values, it represents a composite, assay-derived index rather than an independent analytical endpoint. This distinction is illustrated by Cihan et al. (2025), who found that brewed black tea had significantly higher total antioxidant and total oxidant capacities than brewed green tea, whereas their OSI values did not differ significantly [30]. Thus, differences in both components do not necessarily translate into a corresponding difference in their ratio. A similar distinction was evident in the present study: TAS and TOS differed significantly across flavor categories, whereas OSI did not; in contrast, differences in OSI across preparation media and among the combined temperature–preparation medium groups showed large effect sizes (Table 5). Across the preparation conditions, TAS showed comparatively limited variation, whereas TOS varied more markedly. Accordingly, the OSI pattern appeared to parallel that of TOS more closely, particularly in the almond- and oat-beverage preparations. Nevertheless, because OSI is jointly determined by TAS and TOS, it should be interpreted as a derived index reflecting their relative relationship under the specified experimental conditions, rather than as independent evidence of a particular oxidative mechanism.

From an applied perspective, water-based preparations exhibited the highest measured TAS, whereas preparations at 80 °C was associated with higher TOS and OSI values in the almond- and oat-beverage matrices. Thus, a higher preparation temperature did not consistently produce a more favorable assay-derived oxidant–antioxidant profile across the different matrices. However, these in vitro findings do not establish the physiological superiority of, or potential harm from any preparation medium, nor do they support a specific consumer recommendation. Rather, they should be interpreted as matrix- and assay-dependent differences in the measured chemical profiles of commercial matcha beverages.

### Strengths and Limitations

A major strength of this study was the evaluation of 26 commercial matcha products, unflavored and representing eight flavors under eight standardized preparation conditions involving four preparation media and two temperatures. The joint assessment of TAS and TOS, together with their derived OSI, enabled a more comprehensive interpretation of the assay-detectable oxidant–antioxidant profile, while the use of commercially available products enhanced the real-world relevance of the findings and captured heterogeneity in formulation and labeling.

The findings should be interpreted in light of several limitations. The study was based on in vitro colorimetric measurements and therefore reflects assay-detectable oxidant and antioxidant capacities rather than catechin bioaccessibility, systemic bioavailability, physiological oxidative stress, or health effects. Sample color, turbidity, particulate content, and other matrix-related characteristics may have influenced the TAS and TOS measurements. Although the automated TOS procedure incorporated pre-reaction background correction and bichromatic measurement with a secondary wavelength to minimize intrinsic sample absorbance, no additional dilution-linearity, recovery, or matrix-specific validation experiments were performed for the complex beverage matrices evaluated in this study. Therefore, residual matrix-related interference cannot be completely excluded, particularly for strongly pigmented commercial formulations. Because OSI was derived from TAS and TOS, any matrix-related measurement effects may also have been reflected in this index. Accordingly, the findings for commercial products categorized as beetroot-flavored should be interpreted descriptively. Medium-only controls were not included; therefore, the intrinsic TAS and TOS contributions of the preparation media could not be separated from the responses measured in the complete matcha beverages. Furthermore, each preparation-medium category was represented by a single commercial product, and the plant-based beverages contained additional formulation components. Accordingly, preparation-medium differences should be interpreted as differences in the overall assay responses of the specific commercial beverage formulations evaluated and should not be generalized to cow’s milk, almond beverages, or oat beverages as entire food categories. The use of commercial products introduced substantial formulation heterogeneity. For eight flavored products, the proportion of matcha was not declared, whereas among quantitatively labeled products the matcha content ranged from 80.0% to 99.5%. The amounts and chemical composition of the flavoring ingredients were not independently determined, and the commercial preparation media were characterized on the basis of available label information rather than independent chemical analyses. In addition, flavor categories contained small and unequal numbers of independent commercial products, and flavor category was not experimentally manipulated. Because the products differed simultaneously in brand, matcha proportion, flavoring concentration, and other formulation characteristics, potential brand- and formulation-related confounding cannot be excluded. Consequently, flavor-category differences, including the lower TAS and TOS values observed in commercial products categorized as beetroot-flavored, cannot be attributed specifically to the flavoring ingredient itself. Although the 0.45-µm filtration procedure was selected to obtain samples compatible with automated analysis while preserving the soluble beverage fraction as much as practicable, the extent of particulate or matrix-associated constituent loss was not quantified. Therefore, the reported TAS, TOS, and OSI values should be interpreted as characterizing the filtered fraction of the prepared beverages rather than the complete matcha suspension as consumed. Because the same commercial products were evaluated repeatedly under multiple preparation conditions, observations across conditions were not fully independent. The nonparametric analyses used in the present study did not explicitly model this within-product repeated-measures structure; therefore, the corresponding statistical comparisons should be interpreted with caution. Finally, because the TOS assay measures aggregate oxidizing capacity rather than individual oxidant species, the specific chemical species responsible for the observed TOS differences could not be identified.

## 5. Conclusions

This study demonstrated that the assay-detectable oxidant–antioxidant profiles of commercial matcha beverages varied according to product flavor category and preparation conditions. Commercial products categorized as beetroot-flavored showed lower TAS and TOS values than the other flavor categories, whereas OSI did not differ significantly by flavor. These differences should be interpreted as characteristics of the commercial formulations evaluated rather than as an independent effect of beetroot flavoring. Preparation medium and temperature were also associated with differences in TAS, TOS, and OSI. Water-based preparations exhibited the highest measured TAS values, whereas almond- and oat-beverage preparations at 80 °C showed higher TOS and OSI values, with the highest values observed in the oat-beverage matrix. These findings indicate that beverage matrix and preparation temperature should be considered when evaluating the assay-detectable chemical profiles of matcha formulations. However, the results are specific to the commercial products, preparation conditions, and colorimetric assays evaluated in this study and do not establish bioavailability, physiological oxidative stress, or health effects. In particular, the higher TOS and OSI values observed in heated almond- and oat-beverage preparations should not be interpreted as evidence that these beverages are physiologically more oxidizing or less healthy. Further studies using standardized formulations, matrix-specific analytical validation, targeted compositional analyses, and in vitro digestion models or human studies are needed to clarify the mechanisms underlying these differences and their practical significance.

## Figures and Tables

**Table 1:** General characteristics of the matcha tea products included in the study according to their label information (*n* = 26).

Flavor Type	Brand	Matcha Percentage (%)	Flavoring Percentage (%)	Natural Flavoring Declaration	Additives	Product Quality Claim	Country of Origin	Packaging Type
**Unflavored**	A	100	0	Not declared	Not present	Not declared	Türkiye	Metal
	B	100	0	Not declared	Not present	Not declared	Türkiye (imported raw material)	Metal
	C	100	0	Not declared	Not present	High quality	Türkiye	Paper
**Lemon**	A	Not specified	Not specified	Not declared	Not present	Not declared	Türkiye	Metal
	B	99.5	0.5	Declared	Not present	Not declared	Türkiye (imported raw material)	Metal
	D	90.0	10.0	Declared	Not present	High energy support	Japan (on behalf of the manufacturing company)	Metal
**Strawberry**	A	Not specified	Not specified	Not declared	Not present	Not declared	Türkiye	Metal
	B	99.5	0.5	Declared	Not present	Not declared	Türkiye (imported raw material)	Metal
	D	90.0	10.0	Declared	Not present	High quality	Japan (on behalf of the manufacturing company)	Metal
**Vanilla**	A	Not specified	Not specified	Not declared	Not present	Not declared	Türkiye	Metal
	B	99.5	0.5	Declared	Not present	Not declared	Türkiye (imported raw material)	Metal
	D	90.0	10.0	Declared	Not present	High energy support	Japan (on behalf of the manufacturing company)	Metal
**Mango**	B	99.5	0.5	Declared	Not present	Not declared	Türkiye (imported raw material)	Metal
	C	Not specified	Not specified	Not declared	Present (tricalcium phosphate)	High quality	Türkiye	Paper
	D	90.0	10.0	Declared	Not present	High energy support	Japan (on behalf of the manufacturing company)	Metal
**Pistachio**	B	99.5	0.5	Declared	Not present	Not declared	Türkiye (imported raw material)	Metal
	E	80.0	20.0	Not declared	Not present	Not declared	Türkiye (domestic and imported raw materials)	Paper
	F	Not specified	Not specified	Not declared	Not present	Not declared	Türkiye (imported raw material)	Metal
**Blackberry**	B	99.5	0.5	Declared	Not present	Not declared	Türkiye (imported raw material)	Metal
	C	90.0	10.0	Declared	Not present	Premium ceremonial	Japan (on behalf of the manufacturing company)	Metal
	D	Not specified	Not specified	Not declared	Present (tricalcium phosphate)	High quality	Türkiye	Paper
**Coconut**	B	99.5	0.5	Declared	Not present	Not declared	Türkiye (imported raw material)	Metal
	C	Not specified	Not specified	Not declared	Present (tricalcium phosphate)	High quality	Türkiye	Paper
	D	90.0	10.0	Declared	Not present	High quality	Japan (on behalf of the manufacturing company)	Metal
**Beetroot**	B	Not specified	Not specified	Declared	Not present	Not declared	Türkiye (imported raw material)	Metal
	D	98.0	2.0	Declared	Not present	Premium ceremonial	Japan (on behalf of the manufacturing company)	Metal

**Table 2:** TAS, TOS and OSI values for matcha teas by flavor type (*n* = 208).

Flavor Type	TAS (mmol/L)		TOS (µmol/L)		OSI	
	$\bar{X}$ ± SD	Min–Max	$\bar{X}$ ± SD	Min–Max	$\bar{X}$ ± SD	Min–Max
**Unflavored**	4.49 ± 0.09 ^a^	4.21–4.58	101.73 ± 73.17 ^a^	26.94–262.20	2.26 ± 1.62	0.60–5.84
**Lemon**	4.51 ± 0.08 ^a^	4.30–4.80	103.08 ± 68.43 ^a^	31.02–218.20	2.29 ± 1.52	0.65–4.86
**Strawberry**	4.46 ± 0.15 ^a^	3.85–4.57	102.27 ± 68.32 ^a^	31.13–220.10	2.28 ± 1.51	0.73–4.92
**Vanilla**	4.43 ± 0.30 ^a^	3.06–4.57	100.47 ± 67.40 ^a^	28.56–225.70	2.25 ± 1.48	0.66–5.03
**Mango**	4.43 ± 0.24 ^a^	3.60–4.57	100.09 ± 73.34 ^a^	24.31–241.49	2.23 ± 1.61	0.55–5.37
**Antep pistachio**	4.41 ± 0.24 ^a^	3.53–4.57	91.73 ± 73.54 ^a^	25.77–220.52	2.06 ± 1.63	0.58–4.90
**Blackberry**	4.41 ± 0.30 ^a^	3.42–4.56	101.31 ± 73.66 ^a^	25.69–219.65	2.27 ± 1.63	0.59–4.91
**Coconut**	4.38 ± 0.32 ^a^	3.11–4.56	96.98 ± 74.71 ^a^	15.77–237.57	2.19 ± 1.67	0.37–5.40
**Beetroot**	2.73 ± 1.20 ^b^	0.99–4.52	25.17 ± 25.37 ^b^	0.66–79.07	1.16 ± 1.05	0.01–3.14
***p***	**<0.001**		**0.001**		0.263	
**Test statistics**	34.894		27.144		10.032	

A value of *p* < 0.05 was considered statistically significant, and such *p*-values are presented in bold. For the Kruskal–Wallis test, the *p*-values in the table indicate whether there are differences among the products. For multiple pairwise comparisons, the Bonferroni-adjusted significance level was calculated by dividing α = 0.05 by the number of pairwise comparisons. Means with different superscript letters within the same column are significantly different. OSI: oxidative stress index; TAS: total antioxidant status; TOS: total oxidant status.

**Table 3:** TAS, TOS and OSI values for matcha teas according to preparation medium and temperature (*n* = 208).

Preparation Medium	Preparation Temperature (°C)	TAS (mmol/L)		TOS (µmol/L)		OSI	
		$\bar{X}$ ± SD	Min–Max	$\bar{X}$ ± SD	Min–Max	$\bar{X}$ ± SD	Min–Max
**Water**	**25 °C**	4.55 ± 0.06 ^a^	4.26–4.58	47.51 ± 22.98 ^a^	1.75–88.67	1.04 ± 0.50 ^a^	0.04–1.94
	**80 °C**	4.53 ± 0.07 ^b^	4.16–4.56	59.00 ± 25.60 ^a^	0.66–104.28	1.30 ± 0.56 ^ab^	0.01–2.29
**Cow’s milk**	**25 °C**	4.29 ± 0.68 ^c^	1.91–4.50	30.44 ± 7.26 ^b^	4.23–40.07	0.70 ± 0.15 ^ac^	0.22–1.02
	**80 °C**	3.79 ± 0.89 ^d^	0.99–4.79	32.38 ± 6.83 ^b^	21.41–51.46	0.94 ± 0.49 ^ac^	0.65–3.14
**Almond milk**	**25 °C**	4.27 ± 0.67 ^c^	1.88–4.50	64.16 ± 24.05 ^a^	7.73–102.79	1.46 ± 0.49 ^ab^	0.41–2.32
	**80 °C**	4.24 ± 0.79 ^ce^	1.29–4.51	157.94 ± 52.97 ^c^	15.77–207.82	3.64 ± 0.94 ^d^	0.37–4.61
**Oat milk**	**25 °C**	4.41 ± 0.36 ^e^	3.04–4.52	154.55 ± 38.30 ^c^	19.14–188.95	3.45 ± 0.78 ^d^	0.63–4.19
	**80 °C**	4.39 ± 0.34 ^c^	3.04–4.50	205.81 ± 41.49 ^d^	70.16–262.20	4.64 ± 0.77 ^e^	2.28–5.84
***p***		**<0.001**		**<0.001**		**<0.001**	
**Test statistics**		135.476		147.860		154.404	

A value of *p* < 0.05 was considered statistically significant, and statistically significant *p* values are presented in bold. Kruskal–Wallis test; the *p* values in the table indicate whether there is a difference between all products. For multiple pairwise comparisons, the Bonferroni-adjusted significance level was calculated by dividing α = 0.05 by the number of pairwise comparisons. Means with different superscript letters within the same column are significantly different. OSI: oxidative stress index; TAS: total antioxidant status; TOS: total oxidant status.

**Table 4:** TAS, TOS, and OSI values of matcha teas prepared under different flavor, preparation temperature, and preparation medium conditions (*n* = 208).

Flavor Type	Preparation Medium	TAS (mmol/L)			TOS (µmol/L)			OSI		
		25 °C	80 °C		25 °C	80 °C		25 °C	80 °C	
		$\bar{X}$ ± SD Min–Max	$\bar{X}$ ± SD Min–Max	*p* ^†^	$\bar{X}$ ± SD Min–Max	$\bar{X}$ ± SD Min–Max	*p* ^†^	$\bar{X}$ ± SD Min–Max	$\bar{X}$ ± SD Min–Max	*p* ^†^
**Unflavored**	Water	4.57 ± 0.006	4.56 ± 0.002	0.050	55.60 ± 29.00	63.74 ± 23.65	0.513	1.22 ± 0.63	1.40 ± 0.52	0.513
		4.56–4.58	4.55–4.56		3.33–87.28	44.27–90.06		0.66–1.90	0.97–1.98	
	Cow’s milk	4.50 ± 0.006	4.30 ± 0.13	0.050	30.48 ± 3.17	37.89 ± 5.56	0.050	0.68 ± 0.07	0.88 ± 0.10	0.050
		4.49–4.50	4.21–4.45		26.94–33.07	33.94–44.25		0.60–0.74	0.81–0.99	
	Almond milk	4.48 ± 0.02	4.50 ± 0.007	0.127	64.56 ± 20.85	173.30 ± 20.73	0.050	1.44 ± 0.47	3.85 ± 0.46	0.050
		4.46–4.50	4.50–4.51		50.60–88.52	152.04–193.45		1.13–1.98	3.37–4.29	
	Oat milk	4.50 ± 0.01	4.49 ± 0.002	0.513	160.19 ± 21.70	228.09 ± 29.56	0.050	3.56 ± 0.49	5.07 ± 0.66	0.050
		4.49–4.51	4.49–4.50		139.26–182.58	209.78–262.20		3.09–4.07	4.66–5.84	
		***p*^‡^ =** 0.075	***p*^§^ = 0.020**		***p*^‡^ = 0.033**	***p*^§^ = 0.016**		***p*^‡^ = 0.033**	***p*^§^ = 0.019**	
**Lemon**	Water	4.57 ± 0.01	4.55 ± 0.001	0.050	64.25 ± 16.51	69.85 ± 22.53	0.827	1.41 ± 0.36	1.53 ± 0.49	0.827
		4.55–4.57	4.55–4.56		48.13–81.12	47.09–92.16		1.05–1.78	1.03–2.02	
	Cow’s milk	4.49 ± 0.01	4.50 ± 0.26	0.513	36.32 ± 3.21	33.86 ± 3.97	0.275	0.81 ± 0.07	0.76 ± 0.12	0.827
		4.48–4.50	4.30–4.80		32.83–39.17	31.02–38.40		0.73–0.87	0.65–0.89	
	Almond milk	4.48 ± 0.01	4.50 ± 0.001	0.050	65.84 ± 27.36	178.05 ± 21.81	0.050	1.47 ± 0.61	3.96 ± 0.48	0.050
		4.46–4.49	4.49–4.50		36.69–90.97	154.41–197.40		0.82–2.03	3.43–4.39	
	Oat milk	4.51 ± 0.006	4.49 ± 0.005	0.050	166.58 ± 14.24	209.90 ± 12.11	0.050	3.69 ± 0.32	4.67 ± 0.27	0.050
		4.50–4.51	4.49–4.50		151.56–179.89	196.00–218.20		3.36–4.00	4.36–4.86	
		***p*^‡^ = 0.027**	*p*^§^ = 0.200		***p*^‡^ = 0.043**	***p*^§^ = 0.019**		***p*^‡^ = 0.043**	***p*^§^ = 0.019**	
**Strawberry**	Water	4.57 ± 0.001	4.55 ± 0.0004	0.050	58.59 ± 19.30	75.30 ± 25.28	0.275	1.28 ± 0.42	1.65 ± 0.55	0.275
		4.56–4.57	4.54–4.55		38.96–77.54	46.79–94.98		0.85–1.70	1.03–2.09	
	Cow’s milk	4.49 ± 0.005	4.16 ± 0.27	0.050	36.21 ± 3.64	34.13 ± 3.13	0.513	0.81 ± 0.08	0.82 ± 0.05	0.827
		4.48–4.49	3.85–4.35		32.83–40.07	31.13–37.38		0.73–0.89	0.78–0.87	
	Almond milk	4.44 ± 0.09	4.50 ± 0.006	0.050	62.32 ± 15.03	179.51 ± 32.99	0.050	1.40 ± 0.33	3.99 ± 0.73	0.050
		4.34–4.49	4.49–4.50		51.08–79.40	143.29–207.82		1.14–1.77	3.19–4.61	
	Oat milk	4.51 ± 0.002	4.48 ± 0.01	**0.046**	168.89 ± 17.07	203.23 ± 16.75	0.050	3.74 ± 0.38	4.53 ± 0.39	0.050
		4.51–4.52	4.47–4.49		149.53–181.76	186.59–220.10		3.31–4.03	4.15–4.93	
		***p*^‡^ = 0.023**	***p*^§^ = 0.016**		***p*^‡^ = 0.033**	***p*^§^ = 0.024**		***p*^‡^ = 0.033**	***p*^§^ = 0.024**	
**Vanilla**	Water	4.57 ± 0.001	4.55 ± 0.001	**0.046**	66.19 ± 22.31	79.24 ± 29.21	0.513	1.45 ± 0.49	1.74 ± 0.64	0.513
		4.56–4.57	4.54–4.55		44.05–88.67	47.16–104.28		0.96–1.94	1.04–2.29	
	Cow’s milk	4.49 ± 0.005	3.89 ± 0.74	0.050	32.65 ± 4.54	33.30 ± 4.33	0.827	0.73 ± 0.10	0.86 ± 0.06	0.275
		4.49–4.50	3.06–4.45		29.60–37.87	28.56–37.05		0.66–0.84	0.82–0.93	
	Almond milk	4.43 ± 0.01	4.50 ± 0.009	0.050	60.92 ± 13.32	181.02 ± 16.80	0.050	1.38 ± 0.30	4.02 ± 0.37	0.050
		4.43–4.45	4.50–4.51		51.44–76.14	168.22–200.04		1.17–1.72	3.73–4.43	
	Oat milk	4.50 ± 0.007	4.49 ± 0.004	0.050	135.41 ± 23.50	215.03 ± 12.13	0.050	3.00 ± 0.52	4.79 ± 0.27	0.050
		4.50–4.51	4.48–4.49		109.07–154.25	201.83–225.70		2.42–3.42	4.50–5.03	
		***p*^‡^ = 0.016**	***p*^§^ = 0.015**		***p*^‡^ = 0.025**	***p*^§^ = 0.016**		***p*^‡^ = 0.025**	***p*^§^ = 0.016**	
**Mango**	Water	4.56 ± 0.001	4.55 ± 0.002	0.050	44.78 ± 15.01	61.08 ± 13.70	0.275	0.98 ± 0.33	1.34 ± 0.30	0.275
		4.56–4.57	4.54–4.55		31.03–60.79	50.56–76.57		0.68–1.33	1.11–1.68	
	Cow’s milk	4.49 ± 0.007	3.85 ± 0.22	0.050	29.50 ± 3.96	28.72 ± 3.82	0.275	0.66 ± 0.09	0.74 ± 0.06	0.275
		4.48–4.50	3.60–4.01		24.92–31.82	24.31–31.15		0.55–0.71	0.68–0.79	
	Almond milk	4.48 ± 0.02	4.50 ± 0.007	0.127	73.19 ± 13.34	169.74 ± 17.31	0.050	1.63 ± 0.30	3.77 ± 0.38	0.050
		4.46–4.50	4.50–4.51		58.68–84.93	153.76–188.13		1.30–1.89	3.42–4.17	
	Oat milk	4.52 ± 0.006	4.50 ± 0.0007	0.050	173.35 ± 21.69	220.34 ± 19.28	0.050	3.84 ± 0.48	4.90 ± 0.43	0.050
		4.51–4.52	4.49–4.50		148.58–188.95	203.74–241.50		3.28–4.19	4.53–5.37	
		***p*^‡^ = 0.025**	***p*^§^ = 0.024**		***p*^‡^ = 0.027**	***p*^§^ = 0.016**		***p*^‡^ = 0.027**	***p*^§^ = 0.016**	
**Pistachio**	Water	4.56 ± 0.002	4.55 ± 0.002	0.050	36.34 ± 2.40	46.66 ± 3.05	0.050	0.80 ± 0.05	1.03 ± 0.07	0.050
		4.56–4.57	4.54–4.55		33.65–38.25	44.52–50.16		0.74–0.84	0.98–1.10	
	Cow’s milk	4.46 ± 0.01	3.87 ± 0.30	0.050	28.62 ± 2.74	27.20 ± 0.92	0.513	0.64 ± 0.06	0.71 ± 0.08	0.275
		4.45–4.47	3.53–4.07		25.77–31.24	26.45–28.24		0.58–0.70	0.66–0.80	
	Almond milk	4.43 ± 0.06	4.38 ± 0.20	0.513	52.76 ± 15.97	160.66 ± 34.16	0.050	1.19 ± 0.37	3.65 ± 0.63	0.050
		4.36–4.48	4.15–4.50		35.93–67.70	123.52–190.72		0.81–1.55	2.98–4.24	
	Oat milk	4.50 ± 0.002	4.49 ± 0.01	0.050	165.33 ± 6.72	216.29 ± 5.92	0.050	3.67 ± 0.15	4.82 ± 0.14	0.050
		4.50–4.51	4.48–4.50		160.58–173.01	209.53–220.52		3.56–3.84	4.66–4.90	
		***p*^‡^ = 0.025**	***p*^§^ = 0.025**		***p*^‡^ = 0.022**	***p*^§^ = 0.016**		***p*^‡^ = 0.022**	***p*^§^ = 0.016**	
**Blackberry**	Water	4.56 ± 0.001	4.54 ± 0.003	0.050	39.62 ± 10.22	60.09 ± 19.14	0.127	0.86 ± 0.22	1.32 ± 0.42	0.127
		4.46–4.57	4.54–4.55		31.18–50.98	40.68–78.95		0.68–1.11	0.90–1.74	
	Cow’s milk	4.47 ± 0.02	3.77 ± 0.55	0.050	32.05 ± 5.35	27.67 ± 2.21	0.275	0.72 ± 0.12	0.74 ± 0.06	0.827
		4.44–4.49	3.42–4.41		26.33–36.93	25.69–30.05		0.59–0.83	0.68–0.80	
	Almond milk	4.45 ± 0.05	4.48 ± 0.05	0.275	82.76 ± 20.61	180.33 ± 28.99	0.050	1.86 ± 0.47	4.02 ± 0.61	0.050
		4.40–4.50	4.42–4.51		61.60–102.79	147.16–200.83		1.38–2.33	3.33–4.45	
	Oat milk	4.51 ± 0.006	4.48 ± 0.007	0.050	174.45 ± 13.89	213.47 ± 8.04	0.050	3.87 ± 0.30	4.77 ± 0.19	0.050
		4.50–4.51	4.47–4.48		160.36–188.13	204.38–219.65		3.56–4.17	4.56–4.91	
		***p*^‡^ = 0.025**	***p*^§^ = 0.024**		***p*^‡^ = 0.024**	***p*^§^ = 0.016**		***p*^‡^ = 0.024**	***p*^§^ = 0.016**	
**Coconut**	Water	4.56 ± 0.001	4.54 ± 0.001	0.050	45.02 ± 26.20	53.68 ± 19.31	0.827	0.99 ± 0.57	1.18 ± 0.42	0.827
		4.56–4.57	4.54–4.55		27.09–75.09	31.54–67.05		0.59–1.65	0.69–1.48	
	Cow’s milk	4.47 ± 0.02	3.75 ± 0.63	0.050	29.67 ± 6.46	40.40 ± 13.46	0.275	0.66 ± 0.15	1.05 ± 0.21	0.050
		4.44–4.48	3.11–4.37		22.23–33.86	25.42–51.46		0.50–0.76	0.82–1.18	
	Almond milk	4.47 ± 0.02	4.31 ± 0.16	0.513	87.09 ± 14.02	124.92 ± 97.63	0.513	1.95 ± 0.32	2.86 ± 2.19	0.513
		4.45–4.49	4.21–4.50		73.94–101.83	15.77–203.91		1.65–2.29	0.37–4.53	
	Oat milk	4.51 ± 0.003	4.43 ± 0.06	0.050	167.44 ± 13.67	227.63 ± 13.19	0.050	3.71 ± 0.30	5.14 ± 0.36	0.050
		4.50–4.51	4.39–4.50		158.87–183.20	212.67–237.57		3.52–4.06	4.72–5.40	
		***p*^‡^ = 0.022**	***p*^§^ = 0.033**		***p*^‡^ = 0.033**	*p*^§^ = 0.075		***p*^‡^ = 0.025**	*p*^§^ = 0.082	
**Beetroot**	Water	4.38 ± 0.16	4.34 ± 0.25	1.000	2.07 ± 0.45	2.50 ± 2.60	1.000	0.05 ± 0.008	0.05 ± 0.06	1.000
		4.26–4.49	4.16–4.52		1.75–2.38	0.66–4.35		0.04–0.05	0.02–0.10	
	Cow’s milk	1.96 ± 0.08	1.15 ± 0.22	0.121	12.44 ± 11.61	26.17 ± 6.73	0.121	0.62 ± 0.57	2.39 ± 1.06	0.121
		1.91–2.02	0.99–1.30		4.23–20.65	21.41–30.93		0.22–1.03	1.65–3.14	
	Almond milk	2.00 ± 0.18	1.59 ± 0.43	0.439	9.98 ± 3.19	31.88 ± 1.38	0.121	0.49 ± 0.11	2.11 ± 0.65	0.121
		1.88–2.13	1.29–1.89		7.73–12.24	30.91–32.86		0.41–0.58	1.65–2.57	
	Oat milk	3.19 ± 0.21	3.25 ± 0.30	1.000	41.66 ± 31.85	74.61 ± 6.30	0.121	1.27 ± 0.91	2.29 ± 0.02	0.121
		3.04–3.34	3.04–3.47		19.14–64.18	70.16–79.07		0.63–1.92	2.28–2.31	
		***p*^‡^ =** 0.112	*p*^§^ = 0.104		***p*^‡^ =** 0.160	*p*^§^ = 0.104		***p*^‡^ =** 0.160	*p*^§^ = 0.261	

A value of *p* < 0.05 was considered statistically significant, and statistically significant *p* values are presented in bold. The *p*^†^ values indicate the comparison between 25 °C and 80 °C within the same flavor and preparation medium. The *p*^‡^ values indicate comparisons among the four preparation media (water, cow’s milk, almond milk, and oat milk) at 25 °C within the same flavor. The *p*^§^ values indicate comparisons among the four preparation media at 80 °C within the same flavor. Thus, *p*^†^ represents the comparison between preparation temperatures, whereas *p*^‡^ and *p*^§^ represent comparisons among preparation media at 25 °C and 80 °C, respectively. The Kruskal–Wallis test was used for comparisons involving more than two groups, whereas the Mann–Whitney U test was used for pairwise comparisons. For multiple pairwise comparisons, the Bonferroni-adjusted significance level was calculated by dividing α = 0.05 by the number of pairwise comparisons. OSI: oxidative stress index; TAS: total antioxidant status; TOS: total oxidant status.

**Table 5:** Differences in TAS, TOS, and OSI according to temperature, flavor type, preparation medium, and combined grouping variables.

		TAS	TOS	OSI
**Temperature**	**U**	4440	3968	3552
	***p***	**0.026**	**<0.001**	**<0.001**
	r_rβ_	−0.179	0.266	0.343
**Flavor type**	**χ^2^**	34.9	27.1	10.0
	**df**	8	8	8
	***p***	**<0.001**	**<0.001**	0.263
	**ε^2^**	0.1686	0.1311	0.0485
**Preparation medium**	**χ^2^**	115	132	132
	**df**	3	3	3
	***p***	**<0.001**	**<0.001**	**<0.001**
	**ε^2^**	0.557	0.637	0.638
**Combined temperature–flavor type groups**	**χ^2^**	41.2	38.5	29.7
	**df**	17	17	17
	***p***	**<0.001**	**0.001**	**0.020**
	**ε^2^**	0.199	0.186	0.144
**Combined temperature–preparation medium groups**	**χ^2^**	135	148	154
	**df**	7	7	7
	***p***	**<0.001**	**<0.001**	**<0.001**
	**ε^2^**	0.654	0.714	0.746
**Combined preparation medium–flavor type groups**	**χ^2^**	150	161	152
	**df**	35	35	35
	***p***	**<0.001**	**<0.001**	**<0.001**
	**ε^2^**	0.723	0.780	0.733

A *p* value < 0.05 was considered statistically significant; such *p* values are presented in bold. The Mann–Whitney U test was used for comparisons across temperatures, and the rank-biserial correlation (r_rβ_) was reported as the effect size. The Kruskal–Wallis test was used to compare groups defined by flavor type, preparation medium, and their combinations, and epsilon-squared (ε^2^) was reported as the effect size. Red indicates a large/strong effect; yellow indicates a moderate effect; green indicates a small/weak effect. OSI, oxidative stress index; TAS, total antioxidant status; TOS, total oxidant status.

## Data Availability

The data that support the findings of this study are available from the corresponding author upon reasonable request.

## Abbreviations

The following abbreviations are used in this manuscript:

OSI	oxidative stress index
TAS	total antioxidant status
TOS	total oxidant status
